# New Aspects of HECT-E3 Ligases in Cell Senescence and Cell Death of Plants

**DOI:** 10.3390/plants8110483

**Published:** 2019-11-08

**Authors:** Wei Lan, Ying Miao

**Affiliations:** Fujian Provincial Key Laboratory of Plant Functional Biology, College of Life Sciences, Fujian Agriculture and Forestry University, Fuzhou 350002, China; 1150538001@fafu.edu.cn

**Keywords:** HECT-type E3, cell senescence, aspects, *Arabidopsis*

## Abstract

Plant cells undergo massive orderly changes in structure, biochemistry, and gene expression during cell senescence. These changes cannot be distinguished from the hydrolysis/degradation function controlled by the ubiquitination pathway, autophagy, and various hydrolases in cells. In this mini-review, we summarized current research progress that the human HECT (homologous to the E6AP carboxyl terminus)-type ubiquitin E3 ligases have non-redundant functions in regulating specific signaling pathways, involved in a number of human diseases, especially aging-related diseases, through the influence of DNA repair, protein stability, and removal efficiency of damaged proteins or organelles. We further compared HECT E3 ligases’ structure and functions between plant and mammalian cells, and speculated new aspects acting as degrading signals and regulating signals of HECT E3 ligase in cell senescence and the cell death of plants.

## 1. Introduction

Cell senescence is the final step of organ development, which is a specific form of programmed cell death (PCD) in plants. Cell senescence exists somewhere and sometime during plant development, including the death of root-cap cells, aerenchym formation following hypoxia, senescence of leaves and flowers, leaf sculpturing, terminal treachery element differentiation, tapetal layer degeneration, floral organ abortion, megaspore abortion, degeneration of the suspensor, aleuronat degeneration, and localized cell death following the assault of the pathogen [1]. Cell senescence is triggered by internal and external factors, which is controlled by genetic materials and characterized by degradation and remobilization of cell material to growing tissues and organs. The main and visible changes in plant senescence is the yellowing of leaves, which is a result of chloroplast damage. In fact, besides the damage of the chloroplast, the other organelles have a great alteration at the molecular level and structural level. All of these occur 30% at the transcriptional regulation level and 25% at the macromolecule degradation level in *Arabidopsis* leaf senescence [2,3]. The effective degradation of the macromolecule is important for the senescence process and successful reproduction. Therefore, the ubiquitination pathway, autophagy, and various hydrolases in cells play major roles in cell senescence. Although 26S proteasome-related protein degradation has been well reported, there are still many questions left to be answered regarding the function of ubiquitination in cell senescence. In fact, the ubiquitination not only involves the degradation of substrates, but also acts as a post-translational modification to regulate the activity of substrates, such as histone ubiquitination in mammalian cells [4].

As one component of ubiquitination pathway, the Homologous to the E6AP Carboxyl Terminus (HECT)-type E3s have been implicated in a wide variety of cellular processes including gene expression, nuclear organization, DNA repair, epigenetic modification, and chromosomal stability in animals, which lead to the development of diseases and cancers [5,6]. The HECT E3s family is one of ubiquitin ligases (E3s) family in all eukaryotes, which is characterized by having a C-terminal HECT domain [7]. Although the mechanisms of HECT E3s in animal cells have been revealed, it is still rarely reported in plants. Compared with human HECT E3s, *Arabidopsis thaliana* HECT E3s family has only seven members, which have been divided into five subfamilies, and all of them have their own counterparts (that have the same ancestors, and a similar N-terminal domain) in human HECT E3s [8,9]. Therefore, in this case, we summarize research progress of human HECT E3s in the multiple-physiologically procedure of mammalian cells, including their physiological functions. Regarding the mechanism of substrate recruitment, and regulation of their catalytic activity, we compare HECT E3s ligases’ structure and functions in plant and mammalian cells. Lastly, we speculate the new aspects working as a degrading signal and a regulating signal of HECT E3 ligase in cell senescence and cell death of plants.

## 2. The HECT E3s Family in Plants and Human Being

Similar to the Really Interesting New Gene (RING) E3s family, the HECT E3s widely exist in all organisms, including in animals and plants. There are 28 members of HECT E3s in human beings and seven members in Arabidopsis thaliana [5,6,8]. According to their sequences and protein structures, human HECT E3s are divided into three subfamilies, while there are six subfamilies in plants (five of them exist in Arabidopsis thaliana) (Table 1) [9]. Except for the HECT domain, there are various domains in the N-terminal of HECT E3s, such as the regulator of chromatin condensation 1(RCC1)-like domains (RLDs) in the HECT and the regulator of chromatin condensation 1(RCC1)-like (HERC) domain subfamily, C2 and WW domain in the neural precursor cell expressed developmentally down-regulated protein 4 (Nedd4) family, and a variety of domains in the N-terminal of the other HECTs family. The plant HECT E3s (also called Ubiquitin Protein Ligases (UPLs) family have the same sequence module. It consists of various domains in the N-terminal determining the specific recognition and conjugation of substrates, and the HECT domain in the C-terminal contained the activated cysteine residue (Table 1).

According to previous studies [8,9], there are five subfamilies in Arabidopsis thaliana, including UPL1/UPL2 (subfamily V), UPL3/UPL4 (subfamily I), UPL5 (subfamily VI), UPL6 (subfamily III), and UPL7 (subfamily II) subfamilies, which have counterparts in animals, except UPL5. There are similar N-terminal domains, which are involved in the specific recognition and conjugation of the substrate and involved in the type of ubiquitin chain, between the counterparts, such as the ubiquitin-associated (UBA) domain (for plant UPL1/UPL2 and animal HECT, UBA and WWE Domain Containing 1 (HUWE1), the armadillo (Arm) repeats (for plant UPL1/UPL2/UPL3/UPL4 and animal thyroid hormone receptor interactor 12 (TRIP12 or ULF)), and the isoleucine-glutamine (IQ) motif (for plant UPL6/UPL7 and UBE3B/3C) [8]. It supports the structural basis for the similar function and mechanism between plant and animal HECT E3 ligases [9,10].

## 3. The Patterns of HECT E3s’ Substrate Recruitment and Catalytic Activity Regulation

**Figure 1:**
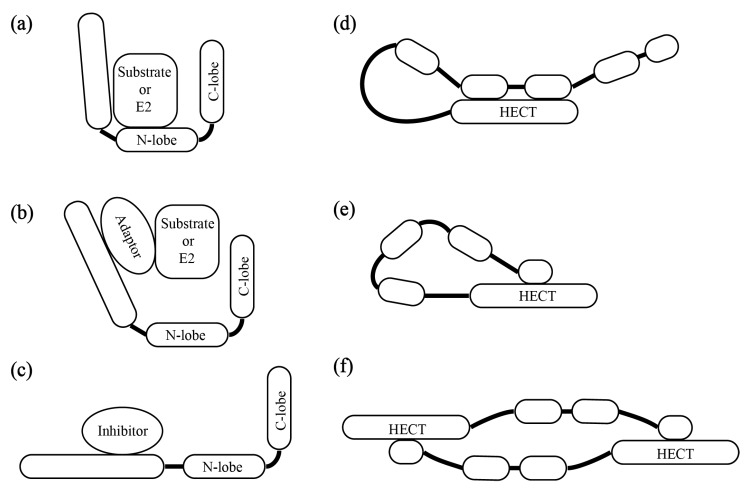
The pattern of HECT E3s’interaction. (**a**). the model that HECT E3s directly interact with substrate or E2 (**b**). The model that the adaptor recruits HECT E3s to its substrate or E2 (**c**). The model that the inhibitor inhibits HECT E3s to interact with substrate or E2 (**d**,**e**). The model of HECT E3s intramolecular interaction (**f**). The model of HECT E3s intermolecular interaction. E2, ubiquitin-conjugating enzymes. N-lobe, the region that locate in the N-terminal of HECT domain. C-lobe, the region that locate in the C-terminal of the HECT domain. HECT: HECT (homologous to the E6AP carboxyl terminus) domain. E2: Ubiquitin-conjugating enzymes, E3: Ubiquitin ligase.

As we knew, the ubiquitination pathway mainly transfers the activated ubiquitin to the corresponding target protein through three significant enzymes: E1, E2, and E3 [11]. The specificity of ubiquitination is mainly determined by ubiquitin-protein E3 ligases (E3s). In the third step of ubiquitination, there are some questions that need to be answered, including how the E3s specifically recognize the substrates, how substrates are recruited to E3s, how E3s control their catalytic activities during ubiquitination, and how E3s determine what kind of ubiquitin chain needs to be formed. At this point, RING E3s do not have catalytic activity, but are able to assist the substrate to be ubiquitinated by E2s, acting as allosteric activators of E2s [12]. On the contrary, HECT E3s have a conservative C-terminal HECT domain, which consist of the N-lobe and C-lobe (where a catalytic cysteine residue is located) [13]. It can form an E3~Ub intermediate product by the catalytic cysteine residue, and then transfer ubiquitin to the substrate [14]. Therefore, HECT E3s are directly involved in the homo-linkage-type of the ubiquitination chain, mono-ubiquitination, or various linkage polyubiquitination. Additionally, there are various domains in the HECT E3s N-terminal, which not only determines its subcellular localization and specific recognition of substrates, but also involves substrate recruitment, catalytic activity regulation, and intermolecular and intramolecular interactions of HECT E3 ligases [15]. The mechanism of interaction between the substrate and HECT E3s not only simply adapt direct binding (Figure 1a), it also provides various ways to regulate HECT E3s’ recruitment and catalytic activity (Figure 1a–f) [15].

The prominent mechanism that regulates the specific recognition and conjugation of substrate is completed by adaptor proteins, which interact with HECT E3s and recruit E3s to their substrates (Figure 1b). For example, the E6 oncoprotein is a classic HECT adaptor protein in mammalian cells, which can interact with E6AP ligase and recruit E6AP to p53 for ubiquitination and degradation. Previous studies showed that E6 bound to the LxxLL motif was located in the N-terminal domain of E6AP and utilized the activity of HECT domain to ubiquitinate p53, which made p53 degradation by the 26S proteasome [16,17]. Individual E6AP or E6 cannot bind to p53. It reveals that the adaptor protein acts as an allosteric activator of HECT E3s, supporting the function of their recognition and interaction [18].

HECT E3s not only utilize the adaptor protein to recruit substrate to E3s, but also recruit E2s to E3s (Figure 1b). Meanwhile, there are inhibitors that prevent HECT E3s for interacting with E2s (Figure 1c). For example, SMAD7 (mothers against DPP homolog 7), as an adaptor for SMURF1 (Smad Ubiquitylation Regulatory Factor 1) and SMURF2, interacts with the WW domains of the SMURF E3s, and recruits these E3s to their substrate for ubiquitination and degradation [19,20]. On the other hand, the E2-binding domain of SMURF2 has an inherent low affinity for its E2s, UbcH7 (ubiquitin conjugating enzyme E2 L3). To facilitate the UbcH7-SMURF2 interaction, the adaptor protein SMAD7 binds to the HECT domain of SMURF2 and the N-terminal domain of UbcH7 [21]. To prevent the E2-E3 interaction, ISG15 (a ubiquitin-like protein) acts as an inhibitor of NEDD4.1, and then reduces ubiquitination of the Ebola virus VP40 (the viral protein), which blocks viral budding [22,23,24,25,26].

HECT E3s are often regulated by intramolecular or intermolecular interactions (Figure 1d–f). Some HECT E3s control the activity of their own catalytic domain through the intramolecular interactions between the N-terminal region and the HECT domain (Figure 1d,e). This action makes the HECT E3s stay in a default state of auto-inhibition. The typical examples are atrophin-1 interacting protein 4 (AIP4) or ITCH (due to an itchy phenotype) and SMURF2, which belong to the NEDD4 subfamily. They consist of the C2 and WW domain in the N-terminal region, and the HECT domain in the C-terminal domain. The WW2-WW3 domain of ITCH interacts with its own HECT domain and then blocks access to the catalytic site [27,28]. In the default state of SMURF2, the C2 domain offers the surface for an intramolecular interaction with the HECT domain [28]. On the other hand, although the same subfamily member, SMURF1, has C2 and WW domains, they adapt intermolecular interactions to control their own catalytic domains (Figure 1f) [29]. We found that the Arm domain of AtUPL3 in *Arabidopsis* can bind its Arm domain to the yeast two hybrid system, which may support the finding that the *Arabidopsis thaliana* HECT E3s also adapt the intermolecular interactions between UPL3s or among UPL3, UPL4, UPL1, or UPL2 including which of them has the Arm domain in the N-terminal region, to affect its catalytic activity. The counterparts of UPL1/UPL2 in animals, HUWE1, showed that they can form an auto-inhibitory homodimer where both intermolecular and intramolecular interactions are involved in the inhibition [30]. Whether this auto-inhibitory homodimer existed in UPLs in *Arabidopsis* remains to be further confirmed.

## 4. The Roles of HECT E3s in Cell Senescence/Aging

### 4.1. The Homeostasis of Substrate Proteins

The main function of ubiquitination is to maintain the homeostasis of substrate proteins by the precise degradation of misfolded protein and short-lived proteins to provide a sound atmosphere for the normal physiological activity and the growth, development, and senescence/aging of organisms [31]. Up to 50% of the total proteins is turned over in plants every week [32]. In mammals, the disorder of homeostasis of substrate proteins may lead cells to abnormal amplification, and may become cancer cells (a state of the cells that broke away from senescence/aging). Thus, the human E3s-deficient cells (HECT E3s mutants are no exception) often cause cancer-prone syndromes. The HECT E3s mutations lead to cervical cancer, Angelman syndrome (AS), and lung cancer due to problems of the DNA damage response, transcription, translation, cell proliferation, apoptosis, and cell differentiation [5,6,33].

**Figure 2:**
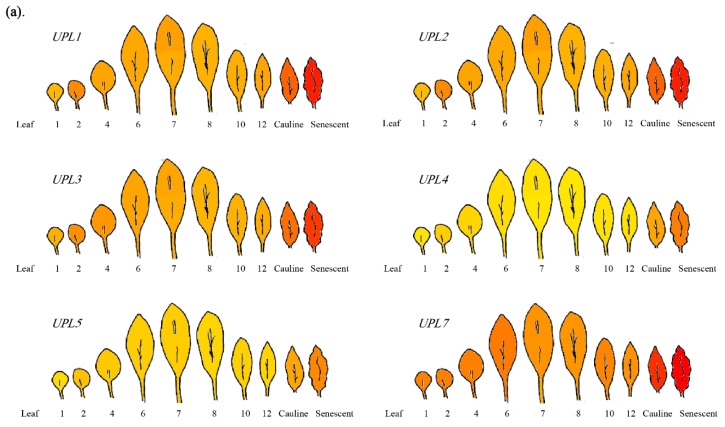

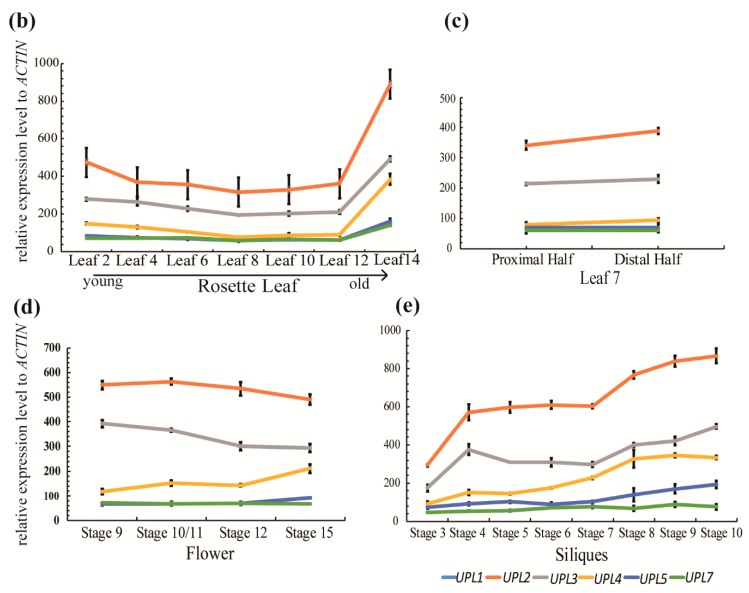
The expression pattern of Ubiquitin Protein Ligases (UPLs) in *Arabidopsis thaliana* from *Arabidopsis* common eFP Browser data (https://bar.utoronto.ca/efp/cgi-bin/efpWeb.cgi) of The *Arabidopsis* Information Resource (TAIR). The eFP Browser by B. Vinegar, drawn by J. Alls and N. Provart. Data from Gene Expression Map of *Arabidopsis* Development [34]. (**a**) The expression pattern of UPLs in leaves. (**b**–**e**) The expression level of UPLs in rosette leaf (from 2nd to senescent leaf) (**b**), in the 7th leaf (**c**), at different stages of flowering (**d**), and seeds (**e**), respectively.

In *Arabidopsis*, based on Arabidopsis eFP Browser dataset (https://bar.utoronto.ca/efp/cgi-bin/efpWeb.cgi) taken from The *Arabidopsis* Information Resource (TAIR), all of the *Arabidopsis* HECT E3s members have the same expression trend, which are highly expressed in senescent leaves (Figure 2a,c). In addition, the HECT E3s’ expression level is higher in the distal half (the elder half) than the proximal half (the younger half) of the 7th of rosette leaves (Figure 2b). Meanwhile, they are highly expressed in seed stages 8–10 of siliques, dry seed, and shoot apex-inflorescence (Figure 2e). These results hint that they are involved in the development, maturity, and production of seeds. Thus, they are supposed to be involved in the remobilization of nutrients from leaves to reproductive tissues and promote its maturity. However, the expression levels of UPL1/UPL2/UPL3 are downregulated, and those of UPL4/UPL5 are slightly upregulated during the maturity of flowers (Figure 2d). These phenotypes suggest *Arabidopsis* HECT E3s play a role in the homeostasis of proteins in various organs and at different development stages, especially the stages of active-protein turnover.

So far, it has been reported that plant UPLs, especially UPL3 and UPL5, are involved in trichrome development, leaf senescence, vascular development, seed size, crop yields, and immunity response [8,35,36,37,38,39]. The *upl5* mutant shows a premature aging phenotype, where the mechanism has been revealed by Ying Miao and Ulrike Zentgraf that UPL5 protein is able to target the transcription factor (TF) WRKY53, which is a key senescence transcription factor, for its ubiquitination and degradation [35]. UPL3 was first reported to play a role in trichrome development [8], despite there being no direct evidence that UPL3 can ubiquitinate GLABROUS 3 (GL3) and ENHANCER OF GL3 (EGL3), which are two bHLH transcription factors that positively regulate the trichrome development and flavonoid biosynthesis in *Arabidopsis*. It can mediate the proteasome-dependent degradation of these two transcription factors [36]. Afterward, the *upl3* mutation shows larger stem diameters than WT. UPL3 may play a role in vascular development [37]. Further findings implicate proteasome-associated HECT-type ubiquitin ligases in the control of plant immune signaling by facilitating substrate polyubiquitination and proteasomal processivity [38]. Recently, Charlotte Miller and colleagues show that UPL3 is involved in LEAFY COTYLEDON2 (LEC2) (a key transcriptional regulator of seed maturation) protein stability, which regulates the seed size and crop yields [39]. In fact, there are other phenotypes in the *upls* mutation, including response to light, drought, biotic stress, etc., but we rarely know their action mechanism (Table 2). Additionally, from the annotations of The *Arabidopsis* Information Resource (TAIR) (https://www.arabidopsis.org/index.jsp), UPLs family members are predicted to be located in almost every subcellular region, including the nucleus (all of the UPLs, UPL1~UPL7), the mitochondrion (UPL1/UPL2), the cytoplasm (UPL1/2/4/5), the plasma membrane (only UPL3), and the plasmodesma (only UPL1). The diversity distribution of UPLs in *Arabidopsis thaliana* reveals that UPLs may contribute to multi-biological pathways.

### 4.2. The Clearance of the Chloroplast or Mitochondrion

Chloroplasts and mitochondria are major sources of reactive oxygen species (ROS) and have been implicated in plant Programmed Cell Death (PCD) regulation, with the latter organelle playing an important role in animal PCD [40]. Therefore, effective removal of damaged chloroplasts and mitochondria is important for preventing Reactive Oxygen Species (ROS)-dependent damage among cells. Moreover, the chloroplasts contain up to 70% of the leaf protein. Therefore, its effective removal promotes the reuse and redistribution of proteins during leaf senescence. Although the mitochondrial integrity and energy status are maintained until the final stages of leaf senescence, the number of mitochondria significantly decreases, and the mitochondria morphology is altered from the elongated, branched structures that are formed from interconnected mitochondria to the enlarged, round-shaped structures [41,42,43]. The removal of the damaged chloroplast and mitochondria is essential for the degradation of macromolecules, which is also defined as protein quality control pathways, such as autophagy, ubiquitination, and the Unfolded Protein Response in the mitochondria (UPRmt) [44,45,46,47]. For example, HUWE1 is involved in mitochondrial clearance by targeting Mitofusin 2 (MFN2), which is an essential component of the mitochondrial outer membrane fusion apparatus, for ubiquitination and degradation. Their interaction can be regulated by Autophagy And Beclin 1 Regulator 1 (AMBRA1), which is a mitophagy receptor for the selective removal of damaged mitochondria in mammalian cells [48,49,50]. Meanwhile, HUWE1 can be located at the mitochondria by interacting with AMBRA1. HUWE1 can also interact with MCL1, which is a potent inhibitor of AMBRA1-mediated mitophagy, for ubiquitination and degradation, which are then involved in mitochondria quality control [51]. These actions demonstrate HECT E3s HUWE1 plays an important role in maintaining the normality of mitochondria. According to information of The Arabidopsis Information Resource (TAIR) (https://www.arabidopsis.org/index.jsp), UPL1 and UPL2 are predicted to be located in the mitochondrion, which may speculate that these two plant HECT E3s may be involved in the degradation of the mitochondrion. Although four UPL members (UPL1, UPL2, UPL4, and UPL5) are located in cytoplasm, they have the probability to translocate to the mitochondria or chloroplast by interacting with the adaptor protein to remove these two organelles.

### 4.3. The Transcriptional Regulation of Senescence-Related Genes via the Chromatin Remodeling and Epigenetic Modification

Besides the degradation functions, the ubiquitination modification also acts as one of epigenetic modification by ubiquitinating the histone or other epigenetic factors. Although, until now, it did not report the changes of histone ubiquitination during plant senescence/aging, it has been known that the histone modifications (including acetylation and methylation) and small noncoding RNAs (ncRNAs) play important roles in leaf senescence [52,53,54]. During cell senescence, most genes undergo up-expression (1432 genes) or down-expression (964 genes), which are regulated by the cross network between chromatin-mediated regulation, transcriptional regulation, posttranscriptional regulation, and post-translational regulation [53,55]. ChIP-seq and RNA-seq data show a significant correlation between histone modifications and gene transcription. Additionally, 786 genes show a significant change of H3K4me3 within the region from the TSS to 500 bp downstream during leaf senescence. Among them, 56% gain the H3K4me3 mark that occurred in senescence upregulation genes, and 63% loss-of H3K4me3 mark occurred in senescence downregulation genes [53]. The key transcript factor WRKY53 suppresses the expression of key negative regulators of senescence by recruiting Histone Deacetylase 9 (HDA9) and a SANT domain-containing protein POWERDRESS (PWR) to its target sites and promoting the removal of H3Ac by HDA9 [56]. Up to date, although there is no evidence showing a correlation between the histone ubiquitination and cell senescence in plants, more studies support histone ubiquitination directly influenced on DNA replication, DNA damage response, gene expression controlling, and DNA/Histone methylation events. All of these may increase the risk of cancer in mammalian cells [57,58,59,60]. The human HECT E3s HUWE1 and HERC2 are involved in histone ubiquitination by directly or indirectly. On the one hand, HUWE1 could directly ubiquitinate histone, while HERC2 can target USP16, RNF8, or RNF168 to influence H2A ubiquitination, which is critical to regulate the DNA damage response [61,62,63,64]. On the other hand, mammalian HECT E3s influence the chromatin stage via ubiquitination and degradation of epigenetic modulators (including histone deacetylase and the ubiquitin ligase). HUWE1/Mule specifically targets HDAC2 (histone deacetylases 2) for ubiquitination and degradation. Therefore, HUWE1/Mule-deficient cells increase the accumulation of HDAC2, which leads to compromised p53 acetylation and crippled p53 transcriptional activation, accumulation, and the apoptotic response upon DNA damage [65].

### 4.4. E4 Ligase-Like Activity

Except for the ubiquitination substrate by direct degradation when HECT E3s work as E3, the HECT E3s also act as E4s to specially mediate the ubiquitin chain elongation of substrates. Five ubiquitin ligases associated with the mammalian proteasome have been identified, including Ube3a/E6-AP, Ube3c/Hul5, Rnf181, Huwe1, and Ubr4 [66]. These proteasome-associated ubiquitin ligases have two general roles on the proteasome. On the one hand, they could modify the ubiquitinated substrates to elongate Ub chains (define as E4s function). On the other hand, they modify the proteasome to regulate its function. Among these ubiquitin ligases, Ube3c/Hul5, which is one of mammalian HECT E3s, has been confirmed that it can elongate the ubiquitin chains of substrates bound to the proteasome to promote their degradation [67,68]. Meanwhile, Ube3c/Hul5 can also extensively and selectively polyubiquitinate the recognized polyubiquitinated protein 13 (Rpn13), which is a subunit of 19S, when proteolysis is even partially inhibited in cells or purified 26S proteasomes with various inhibitors. Rpn13 functions as ‘receptors’ for Ub chains, which is initially bound by ubiquitinated proteins [69]. Its ubiquitination strongly decreases the proteasome’s ability to bind and degrade ubiquitin-conjugated proteins [66].

Similarly, recent studies in plant HECT E3s show that *upl3* mutants exhibit markedly reduced levels of total cellular polyubiquitination. The ubiquitination of Rpn10, a subunit of 19S in *Arabidopsis* and the other ‘receptors’ for Ub chains [69], is also reduced [38]. This phenomenon suggests that UPL3 may modify the proteasome subunits to regulate its activation, and functions as E4s to mediate the form of polyubiquitination, like the mammalian HECT E3s Ube3c/Hul5. E4s-deficiency can block the polyubiquitination of substrates and then reduce its degradation rate, which may reduce and effectively reuse and redistribute nitrogen.

## 5. Conclusions

Cell senescence is accompanied by changes in transcriptional regulation, histone-associated epigenetic processes, posttranslational modification, and macromolecules/organelles degradation. HECT E3s have a conservative C-terminal HECT domain combined with various domains, which determine the pattern of HECT E3s’substrate recruitment and their catalytic activity, resulting in multiple roles in cell senescence of mammalian and plants. The establishment of HECT E3s action mechanism comprise a complicated network at the protein level to regulate various senescence phenotypes and modulate multiple senescence–associated pathways. 

**Figure 3:**
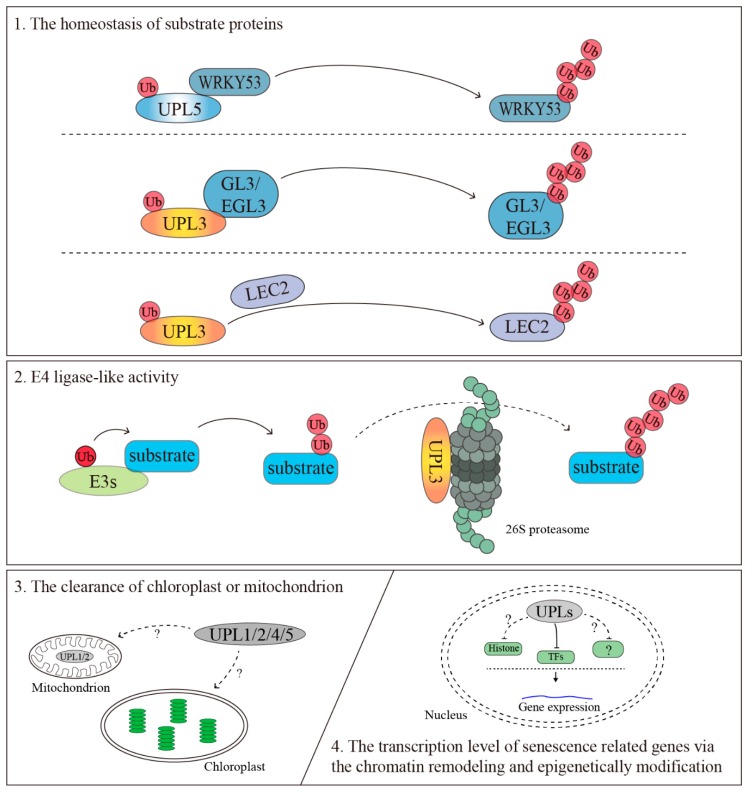
A diagrammatic sketch: the diagrammatic sketch of *Arabidopsis* HECT E3s’ and the hypothesis that HECT E3s regulate plant cell senescence through controlling 1. The homeostasis of substrate proteins. UPL3 and UPL5 involve in plant senescence, trichrome development, and seed maturation via ubiquitinating WRKY53 for degradation [35] and improving the degradation rate of GL3, EGL3, and LEC2 [36,39], respectively. 2. E4 ligase-like activity. Loss of UPL3 markedly reduced the total cellular polyubiquitination and the ubiquitination of Rpn10, which it hints that UPL3 may function as E4s to mediated the elongation of polyubiquitination and the activity of proteasome [38]) 3. The clearance of chloroplast or mitochondrion. Mammalian HECT E3s are involved in maintaining the balance of mitochondria [48,49,50,51] by coordinating with autophagy and unfolding protein. UPL1/2/4/5 are predicted to dually locate in cytoplasm and mitochondrion or plastid, which may involve the clearance of the chloroplast or mitochondrion. 4. Transcriptional regulation via the chromatin remodeling and epigenetically modification. The three members of UPLs protein are also predicted to be located in the nucleus, which may target transcription factors (TFs) [35,36,39], histone, histone deacetylase, etc. to mediate the gene expression [56,59,60,61,62,63,64,65]. E4: A new class of ubiquitylation enzyme, a ubiquitin chain assembly factor.

Revealing the mechanism of cell senescence is important for controlling the cell life span and improving plant biomass yields and organ sizes. HECT E3s play an important role in protein fate and protein function during the senescence process. Although animal studies have shown the diversity of mechanism of HECT E3s functions and catalytic activity regulation, plant HECT E3s’ are still a blank sheet. In this case, based on the understanding of animal HECT E3s function and the existing evidence of plant HECT E3s, we summarize that HECT E3s regulate plant cell senescence by “degradation signal” and “regulation signal” control. 1) The homeostasis of senescence-related substrate proteins by balance of ubiquitin E3s and deubiquitin enzyme (DUBs). 2) E4 ligase-like activity by elongating the ubiquitin chains to proteasome and making proteasome subunit signature. 3) The clearance of the chloroplast or mitochondrion by working as regulatory signal coordination with autophagy or the unfolded protein. 4) Transcriptional regulation of senescence associated genes (SAGs) *via* the chromatin remodeling and epigenetically modification with histone and epigenetic factors (Figure 3). 

With the development of biochemical label-free ubiquitination proteome techniques and phase-separation technique, a global analysis of ubiquitination will illustrate the mechanism of HECT-E3s for the homeostasis of proteins in cells. The phase-separation of complicate protein complexes under various cell environments will address the real-time action module of HECT-E3s in plants. The aspect of the “degradation signal” and “regulation signal” of HECT-E3s in plants may modulate multiple senescence-associated pathways simultaneously and lead to a better control of plant development and plant production biomass and quality. 

## Figures and Tables

**Table 1:** The members of HECT E3s in humans and plants.

Species	Subfamily	Members	Main Domain
In humans (28 members)	HERC family (6 members)	HERC1, HERC2, HERC3, HERC4, HERC5, HERC6	RLDs, HECT domain
	Nedd4 family (9 members)	Nedd4/Nedd4-1, NEDD4L/Nedd4-2, Smurf1, Smurf2, Itch/AIP4, WWP1/AIP5, WWP2, NEDL1/HECW1, NEDL2/HECW2	C2, WW, HECT domain
	other HECTs family (13 members)	HACE1, HECTD1, HUWE1 ^a^, UBE3A/E6-AP, UBE3B, UBE3C, UBR5/EDD1, G2E3, TRIP12, KIAA0317, HECTD3, HECTX/KIAA0614, HECTD2,	ANK, Arm-like, UBA, WWE, IQ, ZnF, PABC, PHD, RING, Filamin, DOC, HECT domain, etc.
In *Arabidopsis thaliana* (7 members)	Subfamily I	UPL3, UPL4	Arm, HECT domain
	Subfamily II	UPL7	IQ, HECT domain
	Subfamily III	UPL6	IQ, HECT domain
	Subfamily V	UPL1, UPL2, UPL8 (lost in *Arabidopsis thaliana*)	Arm, UBA, UIM, HECT domain
	Subfamily VI	UPL5	UBL, C-type lectin, LZ, HECT domain

^a^ The same color was a counterpart in plants and humans. HERC: the regulator of chromatin condensation 1(RCC1)-like domains (RLDs) in the HECT, Nedd4: the neural precursor cell expressed developmentally down-regulated protein 4, SMURF1: Smad Ubiquitylation Regulatory Factor 1, AIP4: atrophin-1 interacting protein 4, UBA: the ubiquitin-associated, WWE: after three of its conserved residues, W and E residues (tryptophans and glutamate respectively), HUWE1: animal HECT, UBA and WWE Domain Containing 1, UBE3: Ubiquitin-protein ligase E3, TRIP12: thyroid hormone receptor interactor 12, ANK: Ankyrin, Arm: armadillo, PHD: plant homeodomain, IQ: isoleucine-glutamine, UIM: Ubiquitin-interacting motif, UBL: Ubiquitin-like domains, LZ: Leucine zipper domain.

**Table 2:** The phenotypes of *upls* mutations and their identified targets.

Mutants	Targets	The Phenotype of Mutants	Reference
*upl3*	^a^ GL3/EGL3 (UPL3-N)	Trichrome development	[36]
*upl3*	Unknown	Larger stem diameters	[37]
*upl3*	LEC2	Larger seed size	[39]
*upl5*	WRKY53	Premature	[35]
*upl1, upl3, upl5*	Unknown	Plant immunity	[38]
*upl3/upl4*	Unknown	Seed germination defect	Unpublished
*upl3, upl3*/*upl4*	Unknown	Light response	Unpublished
*upl2, upl3, upl4, upl6*	Unknown	Plant senescence	Unpublished

^a^ GL3/EGL3: GLABROUS 3 and ENHANCER OF GL3, LEC2: LEAFY COTYLEDON2, UPL3-N: N-terminal of UPL3.
